# Emerging role of Twist1 in fibrotic diseases

**DOI:** 10.1111/jcmm.13465

**Published:** 2018-01-05

**Authors:** Xiaoxuan Ning, Kun Zhang, Qingfeng Wu, Minna Liu, Shiren Sun

**Affiliations:** ^1^ Department of Geriatrics Xijing Hospital Fourth Military Medical University Xi'an Shaanxi China; ^2^ State Key Laboratory of Cancer Biology Fourth Military Medical University Xi'an Shaanxi China; ^3^ College of Life Sciences Shaanxi Normal University Xi'an Shaanxi China; ^4^ Department of Nephrology Xijing Hospital Fourth Military Medical University Xi'an Shaanxi China

**Keywords:** Twist1, epithelial–mesenchymal transition, fibrotic diseases

## Abstract

Epithelial–mesenchymal transition (EMT) is a pathological process that occurs in a variety of diseases, including organ fibrosis. Twist1, a basic helix–loop–helix transcription factor, is involved in EMT and plays significant roles in various fibrotic diseases. Suppression of the EMT process represents a promising approach for the treatment of fibrotic diseases. In this review, we discuss the roles and the underlying molecular mechanisms of Twist1 in fibrotic diseases, including those affecting kidney, lung, skin, oral submucosa and other tissues. We aim at providing new insight into the pathogenesis of various fibrotic diseases and facilitating the development of novel diagnostic and therapeutic methods for their treatment.

## Introduction

Twist1 is a member of the Twist family of basic helix–loop–helix (bHLH) transcription factors that also includes Twist2, Paraxis, Scleraxis, Hand1 and Hand2 in mammals 1. Twist1 was originally discovered in *Drosophila*, and it plays an important role in multiple stages of embryonic development 2. EMT is a vital regulatory mechanism for normal tissue development and the pathological progression of many diseases, including fibrosis and tumour metastasis 3, 4, 5. Apart from serving as a master regulator of embryonic morphogenesis, Twist1 contributes to the EMT process and possesses essential roles in metastasis and fibrosis 6, 7. The role of Twist1 in various cancers has been fully reviewed by Zhu *et al*. 5. In the current review, we discuss the key roles and molecular mechanisms of Twist1 in fibrotic diseases, which may provide new insight into their pathogenesis and treatment.

## Biologic characteristics of Twist1

Twist was originally isolated as a mutant in *Drosophila*, and Twist proteins are evolutionarily conserved from *Drosophila* toother vertebrates 2, 8. In humans, *Twist* gene encodes two isoforms: Twist1 and Twist2. *Twist1* gene is located on chromosome 7p21 and encodes Twist1 protein that consists of 202 amino acids 9. And *Twist2* gene is on chromosome 2q37 and encodes Twist2 protein 10. Both Twist1 and Twist2 are key regulators in embryonic development and organogenesis. A growing number of studies have demonstrated that Twist1 is implicated in many fibrotic diseases 11, 12, 13, while the roles of Twist2 in these diseases are still unexplored. Therefore, this review mainly focuses on Twist1.

*Twist1* gene contains two exons and one intron, with exon1 encoding the protein 9. Twist1 protein contains a typical conserved bHLH motif, which consists of a stretch of basic amino acids followed by two amphipathic α‐helices separated by a loop 14. Twist1 can form functional homodimers or heterodimers with other bHLH factors, including the ubiquitously expressed E‐proteins, and it can bind with *cis*‐regulatory elements called E‐boxes presenting in the promoters of target genes 1, 15. The ratio of Twist1 toother dimer partners is critical for its functions 16. In addition, the phosphorylation of conserved serine and threonine residues within the first α‐helix of Twist1 can affect its dimerization partner choice and transcriptional activity 17. Human Twist1 protein contains two nuclear localization signal (NLS) sequences, and the protein acts as a transcriptional factor in the cell nucleus; however, the translocation of Twist1 with its heterodimer partners is not fully dependent on the NLS sequences 18.

In normal tissues, Twist1 is mainly expressed in mesoderm‐derived tissues, which is consistent with its major role in early embryogenesis, that is, the regulation of mesenchymal cell specification and differentiation 9. During organogenesis, Twist1 partially relies on fibroblast growth factor, bone morphogenetic protein and Shh signalling 16, 19, 20. Twist1 expression is regulated by a large group of upstream regulators or signalling pathways, such as Akt, signal transducer and activator of transcription 3(STAT3), hypoxia‐inducible factor (HIF), nuclear factor (NF)‐κB, SRC (also known as nuclear receptor co‐activator), polyoma enhancer activator 3, distal‐less homeobox gene 4, Wnt/β‐catenin axis, Ras/Msh homeobox protein(MSX2), transforming growth factor (TGF)‐β/Smad/High mobility group A2, and TGF‐β/Fibulin5(for review, see reference 21).

Phosphorylation and ubiquitination are the most important post‐translational modifications that affect Twist1 function by regulating the stability of Twist1 protein. The activation of MAPK can mediate the phosphorylation of Twist1 on Ser68, which inhibits its ubiquitination‐mediated degradation 22. Activation of protein kinase B(AKT1) notably increases Twist1 phosphorylation at Ser42, which is required for Twist1 ubiquitination and degradation 23. Casein kinase 2 can phosphorylate Twist1 at Ser18 and Ser20, leading to prolonged stability of Twist1 24. F‐box and leucine‐rich repeat protein 14 (FBXL14) can mediate and induce the polyubiquitination and degradation of Twist1 25. Histone H3 acetylation on the Twist1 promoter is also an important way to activate Twist1 expression that is not related to stabilize *Twist1* mRNA 26. Although Twist1 promoter hypermethylation is a prevalent event in many tumours, the hypermethylation is not correlated with Twist1 mRNA or protein expression 27, 28.

## The relationship between EMT and fibrotic diseases

During the EMT process, epithelial cells lose their apical–basal cell polarity and cell–cell adhesions and acquire mesenchymal characteristics with a migratory and invasive phenotype 29. Cells lose epithelial markers, such as E‐cadherin and α‐ and γ‐catenin, and obtained mesenchymal cell markers, including fibronectin, vimentin, α‐smooth muscle actin (αSMA), and N‐cadherin 6. According to the biological context, EMT process can be divided into three subtypes 30. Type I EMT plays a critical role in normal embryonic development and organogenesis, which also involves the reverse process, mesenchymal‐to‐epithelial transition (MET). The latter process re‐induces the cells generated by EMT to promote the development of secondary epithelial cells 30. In vertebrates, type I EMT promotes gastrulation and the formation of many tissues and organs, including the heart, neural crest, musculoskeletal system, most craniofacial structures and the peripheral nervous system 31. Type II EMT is implicated in wound healing, tissue regeneration and organ fibrosis, which will be discussed in more detail below 30. Type III EMT occurs in the secondary epithelia of cancerous tissues, and it is involved in cancer cell invasion and metastasis 30.

The EMT program is regulated at multiple levels, and it is modulated mainly by various post‐transcriptional, translational and post‐translational regulators 32, 33. Several transcription factors, including Twist1, Snail family zinc finger 1 (SNAIL1), Zinc finger E‐box‐binding homeobox (ZEB), Prrx1, Klf4, Sox4, Sox9 and others, can trigger the EMT program 34. Twist1 suppresses promoter activity and the expression of E‐cadherin, which is responsible for maintaining the contacts between epithelial cells and for the cell adhesion and relative stability in tissues. The effect of Twist1 on E‐cadherin occurs through binding to the E‐box sequences in the E‐cadherin promoter, which leads to EMT in epithelial cells 6. Twist1 expression is increased in most cancer tissues, and elevated Twist1 expression was found to be directly correlated with the extent of EMT 27, 35.

Fibrosis is initiated by tissue repair programmes and ultimately leads to distorted organ architecture and malfunction 36. This pathological process is characterized by exaggerated deposition of extracellular matrix (ECM), including type I and IV collagen, laminin and fibronectin, which are secreted by fibroblasts/myofibroblasts 37. In healthy tissues, resting fibroblasts maintain homeostasis of ECM components by modulating the synthesis and degradation of ECM 38. Recently, a large body of evidence has shown that the EMT process is involved in organ fibrosis after tissue injury 39, 40. Fibroblasts are activated at the sites of fibrogenesis with increased contractility, and these cells are referred to as myofibroblasts. Although the resident fibroblasts, fibrocytes and pericytes are sources of myofibroblast 41, 42, emerging evidence indicates that the majority of fibroblasts are derived from epithelial and endothelial cells through epithelial/endothelial‐MT (EMT/EndMT) processes defined as type II EMT 38, 43. Type II EMT occurs in the kidney, liver, lung and heart, which are sites closely associated with organ fibrosis 40. Many factors can induce the EMT process resulting in EMT‐related tissue fibrosis, including hypoxia, ECM, cytokines, inflammation and reactive oxygen species, as reviewed by Li *et al*. 39. Hence, targeting the EMT process may provide a potential therapy for fibrotic diseases 39.

## Twist1 and fibrotic diseases

Twist1 is an important transcription factor regulating the EMT process, and its role in fibrotic diseases has been gradually revealed in recent years. However, studies demonstrated that the role of Twist1 in various fibrotic diseases does not align perfectly with the symptoms. Here, we focus on the roles and unique mechanisms of Twist1 in various fibrotic diseases.

### Twist1 and renal fibrosis

Renal fibrosis is characterized by glomerulosclerosis and/or tubular interstitial fibrosis that can be induced in different renal diseases and lead to end‐stage kidney failure 41. EMT contributes to the repair of defective kidney, the activation of renal interstitial fibroblasts, and the deposition of ECM, and the initiation of renal fibrosis 43.

Emerging evidence has shown that EMT transcription factors play important roles in the pathogenesis of renal fibrosis. Boutet *et al*. 44 found that SNAIL1 activation could induce EMT and renal fibrosis in an inducible transgenic mouse. SNAIL1 was reactivated in the murine unilateral ureteral obstruction (UUO) model, an established renal fibrosis model 45, and in the fibrotic lesions from human kidney tissues 44. Inhibition of SNAIL1 expression notably ameliorated established UUO‐induced fibrosis in mice 45. The expression of ZEB1 and ZEB2, which down‐regulation could regulate the expression of epithelial proteins such as E‐cadherin and re‐establish epithelial features 46, was increased in UUO models 47. *In vitro*, suppression of ZEB1 and ZEB2 *via* miR‐200 family reversed TGF‐β1‐induced tubular EMT 48, which may indicate a novel therapeutic strategy for the treatment of renal fibrosis. The activity of transcription factor Snail2/Slug was up‐regulated after UUO and contributed to the EMT process 49. In addition, Slug activation was responsible for TGF‐β1‐induced EMT of renal tubular cells 50.

More recently, the role of Twist1 in renal fibrosis has drawn considerable attention. In 2007, the first study to link Twist with renal fibrogenesis showed that Twist1 expression was elevated in the kidney of UUO mouse models compared with controls. Twist1 was mainly expressed in the tubular epithelia of the expanded tubules and interstitial areas of UUO kidneys 7. In some tubular epithelia, Twist was co‐expressed with fibroblast‐specific protein‐1, a marker for EMT, and in the myofibroblasts located in the expanded interstitial area, Twist colocalized with α‐SMA 7. This study indicated that Twist1 is involved in tubular EMT, myofibroblast proliferation and subsequent fibrosis in obstructed kidneys 7. Lovisa *et al*. 51 found that EMT induced tubular epithelia cell cycle arrest in the G2 phase. They also found that transgenic expression of Twist1 prolonged TGF‐β1‐induced G2 arrest, limiting cellular repair and regeneration. Furthermore, In UUO‐induced renal fibrosis, Twist1 deletion in proximal tubular epithelia cells inhibited the EMT process and maintained the integrity of the cells and restored cell proliferation and the repair and regeneration of kidney parenchyma, ultimately alleviating renal interstitial fibrosis 51. These findings suggest that inhibition of Twist1 represents a potential therapy for renal fibrosis.

During the past few decades, emerging evidence showed that chronic tubulointerstitial hypoxia is one of the final pathways resulting in end‐stage kidney failure in multiple pathological conditions 52, 53. Hypoxia can aggravate the progression of renal interstitial fibrosis in chronic kidney diseases 52, 54. The transdifferentiation of tubular cells into myofibroblasts, which is described as type II EMT induced by hypoxia, is one of the notable causes of kidney fibrosis 55. Following exposure to hypoxia, HIF transcription factors, the well‐known regulators of hypoxia‐adaptive responses, are induced. Structurally, HIFs consist of a hypoxically inducible α subunit, HIF‐1α, HIF‐2α or HIF‐3α, and a constitutively expressed β subunit, HIF‐1β. Under normoxic conditions, the conserved α subunits of HIF are hydroxylated by prolyl hydroxylases (PHDs) using oxygen. The hydroxylated HIF‐α can then be recognized by von Hippel–Lindau protein, which ultimately leads to the ubiquitination and degradation of the modified HIF‐α subunits. In hypoxic conditions, HIF‐α escapes the hydroxylated modification by PHDs because the enzymatic activities of PHDs are inhibited. The unmodified HIF‐α forms a heterodimer with HIF‐1β and then binds to a hypoxia response element (HRE) of its target genes to promote their transcription 54.

**Figure 1:**
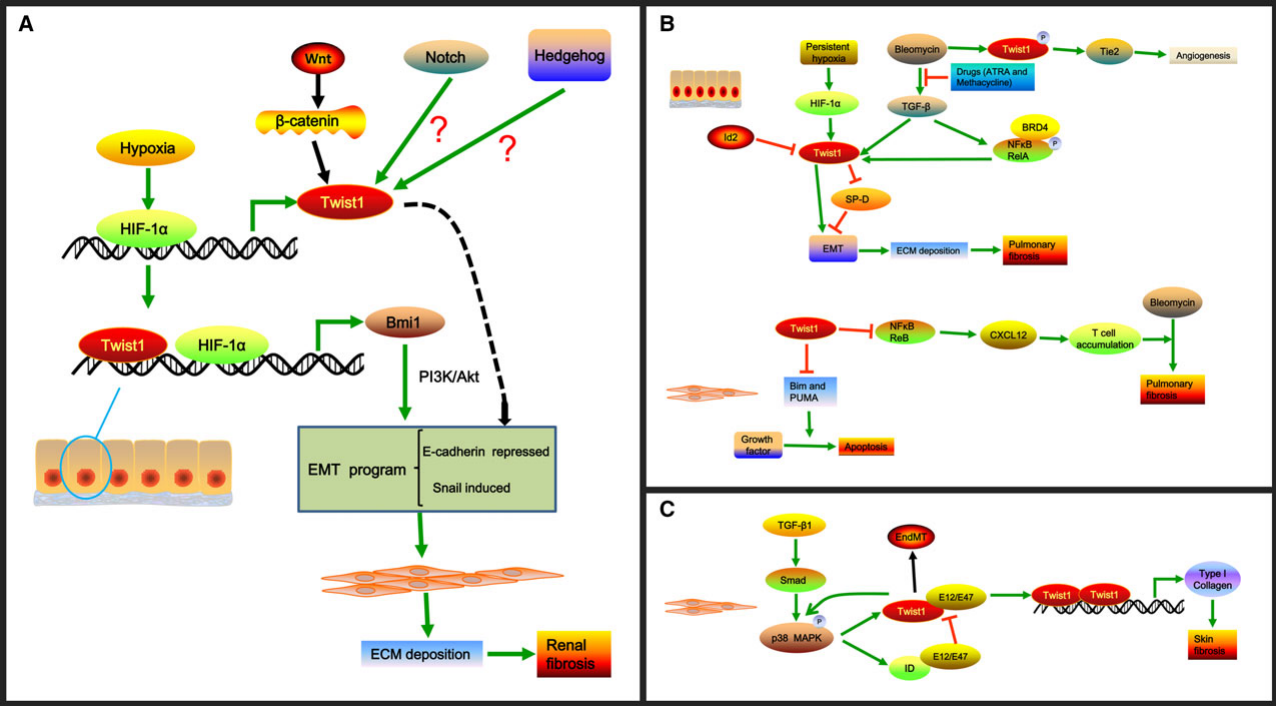
The role and mechanism of Twist1 in renal, pulmonary and skin fibrosis. (**A)** The role and mechanism of Twist1 in renal fibrosis. Hypoxia induces the expression of HIF‐1α that can bind to the proximal HRE of Twist1 at ‐317 to ‐312 in tubular epithelial cells and modulate Twist1 expression. Moreover, Twist1 and HIF‐1α can bind to Bmi1 promoter at ‐732 to ‐727 and ‐190 to ‐185, respectively, and cooperatively promote Bmi1 expression. Bmi1 induces the EMT program *via* activation of PI3K/Akt signalling to increase ECM deposition, resulting in renal fibrosis. The Wnt/β‐catenin pathway can also increase Twist1 expression, leading to renal fibrosis. However, direct interaction between Notch or Hedgehog signalling and Twist1 expression has not been found in renal fibrosis, and the hypothesis needs further investigation. (**B**) The role and mechanism of Twist1 in pulmonary fibrosis. In alveolar epithelial cells, persistent hypoxia induces HIF‐1α expression and *de novo* Twist1 expression, leading to repression of SP‐D that can inhibit the EMT process. Twist1 also promotes the EMT process directly, resulting in the accumulation of ECM and pulmonary fibrosis. Bleomycin‐induced lung fibrosis *via* the activation of TGF‐β1 signalling and up‐regulation of Twist1 could be ameliorated by drugs like ATRA and methacycline that could block TGF‐β1 signalling. Bleomycin‐stimulated Twist1 Ser42 phosphorylation controls angiogenesis *via* activating Tie2 signalling. Id2 could promote the proliferation of primary alveolar epithelial cells and block TGF‐β1‐stimulated type I collagen expression by inhibiting Twist1. BRD4 binds to phospho‐Ser276 NF‐κB/RelA stimulated by TGF‐β1 to regulate the expression of EMT regulators including Twist1. Moreover, Twist1 could protect lung fibroblasts from apoptosis stimulated by growth factor partly by negatively regulating the expression of Bim and PUMA. In addition, loss of Twist1 in collagen‐producing cells augments bleomycin‐induced experimental pulmonary fibrosis that is associated with the elevated expression of non‐canonical NF‐κB transcription factor RelB and T‐cell chemoattractant CXCL12, which causes the accumulation of T cells. (**C**) The role and mechanism of Twist1 in skin fibrosis. Twist1 expression is elevated in fibroblasts of fibrotic skin in a TGF‐β/Smad3/p38‐dependent manner. In turn, the enhanced Twist1 promotes the activation of the p38 pathway. In addition to promoting Twist1 expression, TGF‐β also induces the up‐regulation of ID proteins, which have high affinity for E12/E47 and compete with Twist1 for binding E12/E47 proteins. This situation leads to the formation of Twist1 homodimers that can promote the expression of type I collagen *via* direct binding to the promoters of COL1A1 and COL1A2. Additionally, Twist1 can promote EndMT, which is responsible for skin fibrosis.

Our previous studies have demonstrated that Twist1 plays an important role in hypoxia‐induced EMT in a HIF‐1α‐dependent manner in renal fibrosis 56, 57. Through electrophoretic mobility shift and chromatin immunoprecipitation assays(EMSA), we found that HIF‐1α directly bound to the proximal HRE of Twist1 at ‐317 to ‐312 in tubular epithelial cells and modulated its expression. After hypoxic stimulation, HIF‐1α was found to regulate Twist transcriptional activation and expression, resulting in the promotion of the EMT process 56, 58. Recently, our studies demonstrated that B lymphoma Mo‐MLV insertion region 1 homologue (Bmi1), which is responsible for Twist‐induced EMT in cancer cells 58, is associated with hypoxia‐induced EMT in human tubular epithelial cells and renal fibrosis 59. The Bmi1 promoter contains potential binding sites for HIF‐1α and Twist1, at ‐190 to ‐185 and ‐732 to ‐727, respectively. Hence, under hypoxic condition, HIF‐1α and Twist1 cooperatively promote Bmi1 transcriptional activation and then stabilize its downstream target genes including E‐cadherin and Snail *via* regulation of the PI3K/Akt signalling pathway, leading to renal fibrosis 59 (Fig. 1A).

Wnt signalling proteins play important roles in organogenesis, tissue homeostasis and cancer initiation 60, 61. Wnt proteins transmit signals by binding to the Frizzled receptors and LDL receptor‐related protein co‐receptors. They then dephosphorylate β‐catenin, leading to its stabilization and translocation into the nuclei to stimulate the transcription of the target genes 62. In adult kidney, Wnt signalling is thought to be silenced 63. However, He *et al*. 64 demonstrated that most of the Wnt family members and Frizzled receptors were up‐regulated after UUO treatment. This up‐regulation was accompanied by a remarkable accumulation of β‐catenin in the cytoplasm and nuclei of renal tubular epithelial cells. Furthermore, the expression of Twist1, a potential target gene of Wnt/β‐catenin 65, was significantly elevated, and its expression was closely related to renal β‐catenin abundance. Suppression of the accumulation of β‐catenin notably inhibited Twist1 expression, myofibroblast activation and the expression of fibronectin, fibroblast‐specific protein 1 and type I collagen. Additionally, other studies found that cadmium could induce the transcriptional activation of the Wnt signalling pathway and increase the EMT markers, including Twist1, fibronectin and collagen I, leading to renal fibrosis 66. These findings suggest that Wnt/β‐catenin promotes renal fibrogenesis that is partly dependent on Twist1 activation (Fig. 1A).

Notch and Hedgehog are also two key developmental signalling pathways that are reactivated in the injured kidney and contribute to renal fibrosis 67. In the embryonic mesodermal differentiation process, the regulation of mesodermal cell fate by Notch signalling relied on Twist1 expression 68. Hsu *et al*. 69 showed that Notch1 signalling significantly induced Twist promoter activity and expression related to STAT3 phosphorylation. They further demonstrated that Notch1/STAT3/Twist signalling was correlated with the progression of gastric cancer 69. Kong *et al*. 70 found that the Hedgehog signalling pathway might control Twist1 expression in maintaining a chemoresistant phenotype. Moreover, Twist1 was a direct transcriptional target of Gli, and it might amplify Hedgehog signalling activity and function during limb development 70, which suggests that Twist1 is closely related to the Hedgehog pathway. These results indicate that Twist1 might be regulated by Notch and Hedgehog in renal fibrosis. However, a direct interaction between Notch/Hedgehog and Twist1 in renal fibrosis has not been found, and the hypothesis needs further investigation.

In recent years, Twist1 expression in clinical samples has attracted considerable attention. Our studies showed that Twist1 was rarely expressed in the renal tubules of normal kidneys, whereas it was highly expressed in tubular epithelial cells from the kidneys of patients with chronic kidney diseases 71. Moreover, high expression of Twist1 was associated with the activation of HIF‐1α and the suppression of E‐cadherin 71. Consistent with our study, Liu and co‐workers demonstrated that Twist was not expressed in normal kidneys, whereas activated Twist1 was highly expressed in the tubular epithelial cells from nephrolithiasis patients 67. Twist1 expression was negatively associated with E‐cadherin expression, and Twist1 expression was a critical factor in influencing renal survival in these patients 67. These results indicate that Twist1 expression may be a valuable marker of renal fibrosis progression.

### Twist1 and pulmonary fibrosis

Activated alveolar epithelial cells acquire mesenchymal features that produce mesenchymal proteins, such as laminins and collagens, and cytokines which promote pulmonary fibrogenesis. In addition, active alveolar epithelial cells can induce the proliferation, migration and activation of fibroblasts/myofibroblasts with exaggerated ECM accumulation, resulting in abnormal wound repair 72. Recent studies indicated that during fibrogenesis, phenotypic changes within epithelial cells are modulated by many EMT transcription factors, including Twist1 48, 59, 73.

In recent years, the role of Twist1 in pulmonary fibrosis has begun to be revealed. Veronika *et al*. 74 demonstrated that Twist1 was up‐regulated in lung epithelial cells infected with MHV68, a murine γ‐herpes virus. Its expression was also elevated in type II epithelial cells and fibroblasts in lungs of patients with idiopathic pulmonary fibrosis (IPF) 74, 75. Twist1 levels were also associated with EMT in alveolar epithelial cells 74. Persistent hypoxia, a critical factor contributing to pulmonary fibrosis, induced HIF‐1α expression and *de novo* Twist1 expression, leading to the repression of surfactant protein D (SP‐D), which plays an important role both in innate immunity and acquired immunity and in EMT process (Fig. 1B) 76. In addition, studies have shown that drugs such as all‐*trans* retinoic acid (ATRA) and methacycline could ameliorate bleomycin‐induced IPF *via* suppression of TGF‐β1 signalling and attenuation of Twist1 and Snail expression (Fig. 1B) 77, 78. Furthermore, inhibitor of DNA‐binding 2 (Id2), an inhibitory HLH transcription factor that is highly expressed in lung epithelial cells during development, could promote the proliferation of primary alveolar epithelial cells and block TGF‐β1‐stimulated type I collagen expression by inhibiting Twist expression 79. Id2 was also found to protect mice from pulmonary fibrosis 79. Recent studies showed that BRD4 bound to TGF‐β1 stimulated phospho‐Ser276 NF‐κB/RelA and regulated the expression of EMT regulators Twist1, Snail and ZEB1 80. These results suggest that Twist1 may be an important transcription factor that promotes EMT in the development of pulmonary fibrosis. Apart from the role of Twist1 in EMT, Bridges and co‐workers 75 found that it could protect lung fibroblasts from apoptosis stimulated by growth factors partly *via* negatively regulating the expression of Bim and PUMA, both of which are members of the pro‐apoptotic Bcl‐2 family (Fig. 1B). Moreover, Twist1^Ser42^ phosphorylation controls angiogenesis through angiopoietin‐Tie2 signalling, which contributes to the pathogenesis of bleomycin‐induced pulmonary fibrosis (Fig. 1B) 81. In contrast, a recent *in vivo* study demonstrated that loss of Twist1 in collagen‐producing cells augmented bleomycin‐induced experimental pulmonary fibrosis, which was characterized by a notable accumulation of T cells and bone marrow‐derived matrix‐producing cells. This outcome was associated with an elevated expression of non‐canonical NF‐κB transcription factor RelB and T‐cell chemoattractant CXCL12 (Fig. 1B) 82. These findings were consistent with a previous study showed that Twist1 could suppress NF‐κB‐induced inflammation responses 83. Taken together, these results indicate that Twist1 may promote EMT and inhibit fibroblast apoptosis. Twist1 phosphorylation contributes to the angiogenesis in IPF; However, Twist1 also regulates inflammation in pulmonary fibrogenesis. Hence, the exact role of Twist1 in pulmonary fibrosis needs further study.

### Twist1 and skin fibrosis

Recently, Twist1 was found to function in skin fibrosis. Palumbo‐Zerr *et al*. 13 reported that Twist1 expression was elevated in fibroblasts of fibrotic human and murine skin in a TGF‐β/Smad3/p38‐dependent manner. In turn, the enhanced Twist1 promoted TGF‐β‐induced fibroblast activation, which was related to the activation of the p38 pathway. In addition to promoting Twist1 expression, TGF‐β also increased the level of inhibitor of differentiation (ID) proteins, which had a high affinity for E12/E47 and competed with Twist1 for binding E12/E47 proteins. This situation led to the formation of Twist1 homodimers that could promote the expression of type I collagen *via* directly binding to the promoters of COL1A1 and COL1A2 (Fig. 1C) 13. Furthermore, Palumbo‐Zerr *et al*. 13 found that mice selectively lacking Twist1 in fibroblasts were protected from experimental skin fibrosis and were comparable to mice with ubiquitous inactivation of Twist1. These results suggest that Twist1 functions as a pro‐fibrotic factor in skin fibrosis mainly by activating resident fibroblasts 13.

EndMT is another possible pathogenic mechanism for systemic sclerosis 84. The expression of Twist1, an important EndMT factor, was increased in bleomycin‐induced scleroderma mouse models and cultured human umbilical vein endothelial cells. Geniposide, which has a protective effect on endothelial cells in the bleomycin‐induced scleroderma mouse model, remarkably decreased Twist1 expression 84. This finding indicated that targeting Twist1 could attenuate the EndMT process, leading to the alleviation of skin fibrosis. Taken together, the data suggest that Twist1 not only promotes the activation of resident fibroblasts, but also induces EndMT in skin fibrosis 13, 84.

### Twist1 and other fibrotic diseases

Oral submucous fibrosis is a chronic progressive scarring disease characterized by the accumulation of dense fibrous connective tissue with exaggerated inflammatory cell infiltration and epithelial atrophy in the submucosal layer 12, 85. EMT has been shown to be induced in oral submucous fibrosis 86. Epidemiological evidence indicated that oral submucous fibrosis was closely related to habitual chewing of areca nuts. Arecoline increased Twist1 expression in human primary buccal mucosal fibroblasts, and the myofibroblast activity activated by arecoline could be reversed by the Twist1 knockdown 12. In addition, Twist1 inhibition suppressed fibroblast activation in primary cultivated oral submucous fibroblast cells 12. Twist1 expression was also found to be higher in oral submucous fibrotic tissues in association with areca quid chewing compared to normal oral mucosa tissues 12. These results indicate that Twist1 up‐regulation may contribute to the pathogenesis of oral submucous fibrosis; however, the exact mechanism remains to be determined.

A hallmark of liver fibrosis is the activation of hepatic stellate cells (HSCs), leading to the deposition of collagen protein. Recent studies showed that Twist1 expression and its role in liver fibrosis seem to be contradictory. One study showed that Twist1 expression was increased in fibrotic livers from patients infected with hepatitis C virus (HCV) and in mouse fibrotic livers induced by methionine‐ and choline‐deficient diet treatment as well as in activated primary‐cultured mouse HSCs 87. This study also showed that Twist1 might regulate miR‐214‐5p to activate HSCs and promote the progression of liver fibrosis 87. Another study reported that Twist1 expression was reduced in HSCs of fibrotic livers from carbon tetrachloride‐treated mice or in ethanol‐activated primary HSCs 88. Nanovesicular exosomes containing high levels of Twist1 were secreted by quiescent HSCs but not activated HSCs. Twist1 bound to the E‐box in the miR‐214 promoter and activated its transcription and expression, leading to CCN2 suppression. This study revealed a unique function for cellular or exosomal Twist1 in CCN2‐dependent fibrogenesis 88. The discrepant expression and effect of Twist1 in these studies may result from the use of different inducers.

Twist1 has also been found to play a role in peritoneal membrane (PM) fibrosis. EMT contributes to PM fibrosis as shown by He and co‐workers 11. They found that elevated Twist1 expression was positively correlated with EMT progress and PM fibrosis in patients who underwent continuous ambulatory peritoneal dialysis, in high glucose damage of human peritoneal mesothelial cells(HPMCs), and in peritoneal dialysis rat models. Twist1 bound to YB‐1, resulting in a promotion in EMT, proliferation and cell cycle progression of HPMCs, which might contribute to PM fibrosis 11. These results indicate that Twist1 leads to PM fibrosis during peritoneal dialysis treatment, mainly through regulating YB‐1 expression 11.

EndMT contributes to the development of cardiac fibrosis, which ultimately leads to cardiac remodelling. A direct correlation has not been found between Twist1 and cardiac fibrosis; however, a study by Gong *et al*. 89 found that blocking the TGF‐β1/Smads pathway could suppress the EndMT, which is involved in the inhibition of its downstream transcription factors, including Twist1. This outcome suggests that Twist1 may function in the progression of cardiac fibrosis. EMT of biliary epithelial cells plays a critical role in biliary fibrosis. Simvastatin could prevent biliary fibrosis involved in the EMT progress of biliary epithelial cells that was related to the inhibition of Twist1 expression 90, which indicates that Twist1 may also be a feasible target for biliary fibrosis.

## Conclusions and perspectives

Twist1, a bHLH transcription factor, plays an important role in embryonic development, cancer metastasis and fibrotic diseases, all of which are closely related to EMT progress. Recent studies have shown that Twist1 is abnormally expressed in various fibrotic diseases. Twist1 expression is highly expressed in most fibrotic diseases, including those affecting renal, skin, oral submucous, and PM tissues and biliary fibrosis. Elevated Twist1 may promote the progression of these diseases *via* induction of the EMT/EndMT program. Although Twist1 expression is enhanced in pulmonary fibrosis, and it can promote EMT process of epithelial cells and inhibit the apoptosis of lung fibroblasts in pulmonary fibrosis, *in vivo* studies have found that loss of Twist1 in collagen‐producing cells augments bleomycin‐induced IPF by exaggerating inflammatory response. Recently, the role of Twist1 phosphorylation in angiogenesis in IPF was described 81. Angiogenesis is an important factor contributing to the pathogenesis of many fibrotic diseases 36, 91, 92, hence Twist1 phosphorylation may also be related to fibrotic diseases other than IPF. The expression and role of Twist1 in liver fibrosis are inconsistent in different studies, possibly because different inducers were used. Once the molecular mechanism is better understood, Twist1 may be regarded as a novel therapeutic target for the treatment of multiple fibrotic diseases. Additionally, given that Twist1 expression can be measured in clinical samples, it may serve as a valuable marker in tracking the progression of many fibrotic diseases. In conclusion, the current review comprehensively summarized the role and underlying mechanisms of Twist1 in various fibrotic diseases. This summary may foster a better understanding of this molecular signal and the pathogenesis of fibrotic diseases.

## Conflict of interest

The authors have no conflict of interest to disclose.
